# Structural Analysis of Merkel Cell Polyomavirus (MCPyV) Viral Capsid Protein 1 (VP1) in HIV-1 Infected Individuals

**DOI:** 10.3390/ijms21217998

**Published:** 2020-10-27

**Authors:** Carla Prezioso, Martina Bianchi, Francisco Obregon, Marco Ciotti, Loredana Sarmati, Massimo Andreoni, Anna Teresa Palamara, Stefano Pascarella, Ugo Moens, Valeria Pietropaolo

**Affiliations:** 1 IRCSS San Raffaele Pisana, Microbiology of Chronic Neuro-degenerative Pathologies, 00163 Rome, Italy; carla.prezioso@uniroma1.it; 2 Department of Public Health and Infectious Diseases, “Sapienza” University of Rome, 00185 Rome, Italy; francisco.obregon1703@gmail.com; 3 Department of Biochemical Sciences “A. Rossi Fanelli”, “Sapienza” University of Rome, 00185 Rome, Italy; martina.bianchi@uniroma1.it (M.B.); Stefano.pascarella@uniroma1.it (S.P.); 4 Laboratory of Clinical Microbiology and Virology, Polyclinic Tor Vergata Foundation, 00133 Rome, Italy; marco.ciotti@ptvonline.it; 5 Infectious Diseases Clinic, Policlinic Tor Vergata, 00133 Rome, Italy; sarmati@med.uniroma2.it (L.S.); andreoni@med.uniroma2.it (M.A.); 6 Department of System Medicine, Tor Vergata University of Rome, 00133 Rome, Italy; 7 Department of Public Health and Infectious Diseases, Institute Pasteur, Cenci-Bolognetti Foundation, Sapienza University of Rome, 00185 Rome, Italy; annateresa.palamara@uniroma1.it; 8 IRCCS San Raffaele Pisana, Telematic University, 00163 Rome, Italy; 9 Department of Medical Biology, Faculty of Health Sciences, University of Tromsø, 9037 Tromsø, Norway; ugo.moens@uit.no

**Keywords:** Merkel cell polyomavirus (MCPyV), viral protein 1 (VP1), amino-acids mutation, protein structural organization, HIV+ individuals, A549 culture system

## Abstract

Merkel cell polyomavirus (MCPyV) viral protein 1 (VP1) is the capsid protein that mediates virus attachment to host cell receptors and is the major immune target. Given the limited data on MCPyV VP1 mutations, the VP1 genetic variability was examined in 100 plasma and 100 urine samples from 100 HIV+ individuals. Sequencing of VP1 DNA in 17 urine and 17 plasma specimens, simultaneously MCPyV DNA positive, revealed that 27 samples displayed sequences identical to VP1 of MCC350 strain. VP1 from two urine specimens had either Thr47Ser or Ile115Phe substitution, whereas VP1 of one plasma contained Asp69Val and Ser251Phe substitutions plus deletion (∆) of Tyr79. VP1 DNA in the remaining samples had mutations encoding truncated protein. Three-dimensional prediction models revealed that Asp69Val, Ser251Phe, and Ile115Phe caused neutral effects while Thr47Ser and Tyr79∆ produced a deleterious effect reducing VP1 stability. A549 cells infected with urine or plasma samples containing full-length VP1 variants with substitutions, sustained viral DNA replication and VP1 expression. Moreover, medium harvested from these cells was able to infect new A549 cells. In cells infected by samples with truncated VP1, MCPyV replication was hampered. In conclusion, MCPyV strains with unique mutations in the *VP1* gene are circulating in HIV+ patients. These strains display altered replication efficiency compared to the MCC350 prototype strain in A549 cells.

## 1. Introduction

Merkel Cell Polyomavirus (MCPyV), a member of the *Polyomaviridae* family, possesses a non-enveloped icosahedral capsid that contains a circular double stranded DNA viral genome of approximately 5400 bp divided into the early and the late region [1]. Alternative splicing and alternative use of open reading frames (ORF) generate the proteins large tumor (LT), small T (sT), and 57 kT antigens, and LT open reading frame (ALTO) [2,3,4]. After the initiation of viral DNA replication, the late region, encoding for the structural components of the virus capsid, virus protein 1 (VP1) and the minor capsid protein virus protein 2 (VP2), is transcribed from the opposite direction respective to the early region. Unlike others human polyomaviruses (HPyVs), MCPyV does not seem to express VP3, despite an in-frame internal adenine-thymine-guanine (ATG) start codon in the *VP2* gene [5]. Conversely, the absence of a VP3 ORF seems to be a common feature in the genomes of chimpanzee, raccoon and bat PyVs [5]. HPyV genomes that lack VP3 seem to encode a VP1 protein that is larger than VP1 found in HPyVs with VP3, whereas the VP2 ORF is shorter than in VP3 expressing HPyV species and lacks a predicted nuclear localization signal (NLS). This remark supports the hypothesis that VP1 could be able to absolve the functions of VP3 [5]. VP1 alone or VP1 together with VP2 self-assemble into 45–55 nm diameter virus-like particles (VLPs) that are used in serological assays [6]. The early and late region are separated by the non-coding control region (NCCR) that contains the origin of replication (ORI) and bidirectional transcriptional elements that govern early and late viral gene expression [7]. Rearrangements in the NCCRs of BKPyV and JCPyV affect viral DNA replication, promoter activity, virus production, and could help to increase the pathogenic properties of these viruses [8,9,10]. Polymorphism in the MCPyV NCCR has been observed that influences early and late promoter activity, but the biological implications on the viral life cycle of the virus and the pathogenic properties of MCPyV remain to be investigated [11].

MCPyV is, so far, the first HPyV directly implicated in the development of a human cancer, namely Merkel cell carcinoma (MCC). MCC is a neuroendocrine, cutaneous malignancy that, although rare, is highly aggressive displaying a mortality rate of ~45% [12]. The MCC incidence has increased significantly during the last 10 years and is prospected to escalate further because of ageing of the human population [13,14]. Approximately 10% of all MCC patients presented some form of immunosuppression including HIV-1/AIDS, chronic inflammatory conditions, solid organ transplantation, or hematological malignancies [15]. MCPyV prevalence studies suggest that the virus is chronically shed from human skin representing part of the skin microbiota [16]. The initial exposure to MCPyV, based on the VP1 serology assay, supposedly occurs in early childhood and rapidly increases with age, remaining frequent in adulthood (79–96.2%) [16]. Antibodies versus MCPyV LT and sT are detected in about 1% of healthy individuals, whereas ~50% of MCPyV-positive MCC patients have these antibodies [17]. These patients often have higher titers of VP1 antibodies than normal healthy individuals [6]. Phylogenetic analysis based on the LT amino acid sequence reveals that MCPyV is most closely related to gorilla polyomavirus 1 and chimpanzee polyomaviruses 2 and 3 [18], raising the possibility that MCPyV stems from a non-human primate [18]. MCPyV may have been transmitted from apes to humans by bush meat consumption or direct contact with animals [19].

To date, the events connecting initial MCPyV infection and subsequent cell transformation still remain elusive, although it has been established that viral DNA integration into the host genome and continued expression of the C-terminal truncated LT are required for MCC development [20]. The C-terminus of LT contains anti-tumorigenic properties and may explain why this region is deleted in MCC [12]. In addition to LT truncation, also mutations in VP1 have been proposed to be indirectly involved in viral DNA integration and tumorigenesis [21,22]. As known, VP1 is implicated in the mechanisms underlying protein self-assembly, attachment to host cell receptors, virus entry, immune response and intracellular trafficking and it plays an important role in the diagnosis of HPyVs infection and in epidemiologic studies [23,24,25,26,27]. Baez and colleagues, by an overall analysis of VP1 sequences, showed a higher variability in the N- and C-terminal regions with a characteristic deletion (∆) of six amino acids (18–23) in the N-terminus region (aa 1-51) [21]. Kassem and colleagues, analyzing 39 MCC samples, found a 90 bp loss in the VP1 gene from one MCPyV-positive MCC sample, with an impact on the gene open reading frame [22]. Finally, Mertz and colleagues reported that, in other HPyVs, deletions of genes encoding for structural protein are most likely to occur within the process of viral DNA integration [28]. Nevertheless, these results did not explain the impact of these deletions on the VP1 structure or on the development of MCC. In the light of these considerations, to add new information on this topic, the aim of this study was to investigate the genetic variability of the *VP1* gene in MCPyV strains circulating among a HIV+ population and its potential effect on the three-dimensional structure of the protein. Moreover, to address the consequences of the VP1 mutations, in vitro studies were performed in order to understand whether amino-acids changes in VP1 could have any implications on MCPyV biology. 

## 2. Results

### 2.1. Detection and Quantification of MCPyV DNA in Urine and Plasma Samples by Quantitative Real-Time Polymerase Chain Reaction (qPCR) Analysis

**Figure 1:**
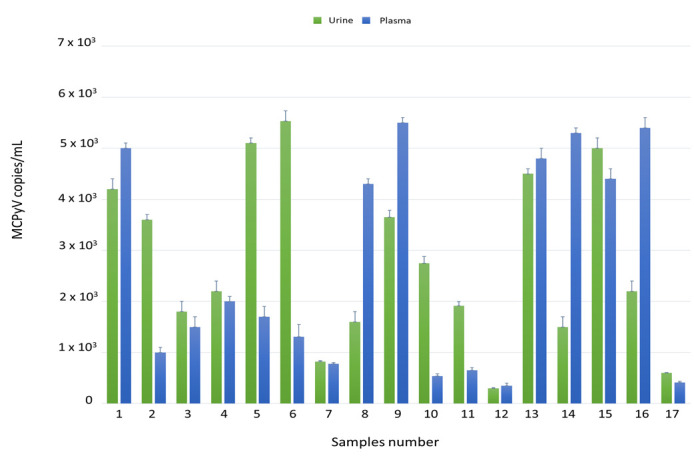
Detection and quantification of Merkel cell polyomavirus (MCPyV) DNA in urine and plasma *ex vivo* samples by qPCR. Data are expressed as the mean of three independent experiments, and error bars indicate standard deviations. MCPyV DNA ranged in urine from 3 × 10^2^ copies/mL in MCPyV 12 to 5.5 × 10^3^ copies/mL in MCPyV 6 and in plasma from 3.5 × 10^2^ copies/mL in MCPyV 12 to 5.5 × 10^3^ copies/mL in MCPyV 9.

Each sample (100 urine and 100 plasma, for a total of 200 specimens) was tested for MCPyV DNA by quantitative real-time polymerase chain reaction (qPCR). Overall, MCPyV DNA was detected in 23/100 (23%) urine and in 17/100 (17%) plasma samples. qPCR results are reported in Figure 1. No significant association was found between the presence of MCPyV DNA and MCPyV viral load versus age, gender, and HIV patient’s status (naïve/experienced) at enrolment time. Finally, detection of MCPyV did not show a correlation with HIV-1 load at enrolment nor with CD4+ cell counts. 

### 2.2. Nucleotide Analysis of MCPyV VP1

**Figure 2:**
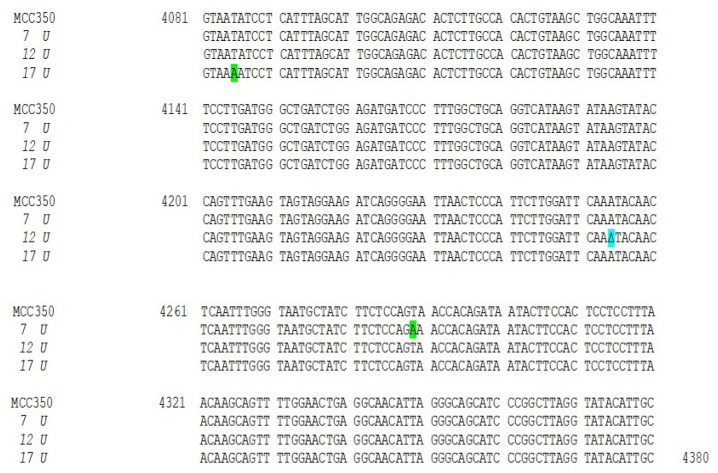
Nucleotide analysis of MCPyV VP1 in urine samples. The alignment is shown between the nucleotide sequence from 4081 to 4380 of the published sequence of MCPyV in GenBank (MCC350) [20] and that obtained from the sequencing of urine positive for MCPyV VP1 (MCPyV 7 U, 12 U, 17 U). Highlighted in green: the 4289 T to A transversion in 7 U and the 4085 T to A transversion in 17 U. Highlighted in blue: the single 4254 ∆ (A) in 12 U.

**Figure 3:**
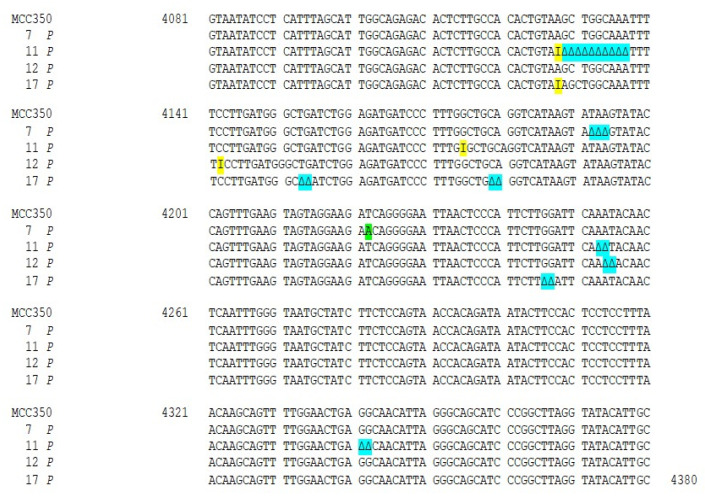
Nucleotide analysis of MCPyV VP1 in plasma samples. The alignment is shown between the nucleotide sequence from 4081 to 4380 of the published sequence of MCPyV in GenBank (MCC350) [20] and that obtained from the sequencing of plasma positive for MCPyV VP1 (MCPyV 7 P, 11 P, 12 P, 17 P). A 4222 T to A transversion was present in 7 P (highlighted in green). Highlighted in blue: a ∆ of a 3 bp (TAA) at nucleotide positions 4192–4194 in 7 P; a 10 bp ∆, encompassing nucleotide position 4128–4137 and two pairs of ∆ at nucleotide positions 4253–4254 (AA) and at nucleotide positions 4341–4342 (GG) in 11 P; a double AT ∆ (position 4254–4255) was identified in 12 P; 3 double ∆, a TG∆ (4153–4154), a CA∆ (4179–4180) and GG∆ a (4246–4247), were revealed in 17 P. Highlighted in yellow: 11 P displayed two pairs of I between 4127–4128 and 4174–4175 nucleotide positions and a 42 bp I was present in 17 P between nucleotides 4127 and 4128.

Thirty-four samples, simultaneously positive to MCPyV DNA detection in urine (MCPyV 1 U–17 U) and in plasma (MCPyV 1 P–17 P) were arbitrarily selected and used for MCPyV VP1 nucleotide analysis, after amplification and sequencing. The amplified VP1 DNA fragments, spanning from nucleotide position 3156 to 4427, were compared with the reference sequence of the prototype North American strain MCC350 [20]. The analysis of 14 VP1 DNA recovered from urine (MCPyV 1 U–6 U, 8 U–11 U, 13U–16U) and 13 plasma (MCPyV 1 P–6 P, 8 P–10 P, 13 P–16 P) specimens, showed identical sequences compared to the prototype strain (data not shown). Interestingly, the remaining MCPyV urine and plasma positive samples (MCPyV 7 U, 12 U, 17 U and MCPyV 7 P, 11 P, 12 P, 17 P), displayed a VP1 nucleotide structure characterized by the presence of several mutations (Figure 2 and Figure 3). In detail, MCPyV 7 present in urine (7 U) contained the 4289 T to A transversion whereas, in plasma (7 P), a 4222 T to A transversion and a ∆ of a 3 bp (TAA) at nucleotide positions 4192-4194 were observed (Figure 2 and Figure 3). MCPyV 11 P displayed two pairs of insertions (I) between nucleotide positions 4127–4128 (ATATTGCCTCCCACATCTGCAATGTGTCACAGGTAATATCCT) and between nucleotide positions 4174–4175 (CATTTAGCATTGGCAGAGACACTCTTGCCACACTGTA). In addition, a 10 bp ∆ (AGCTGGCAAA), encompassing nucleotide position 4128–4137 and two pairs of ∆ at nucleotide positions 4253–4254 (AA) and at nucleotide positions 4341–4342 (GG) were found (Figure 3). Regarding MCPyV 12, in urine sample (12 U) the single 4254 ∆ (A) was present (Figure 2), conversely, in plasma specimen (12 P) a 21 bp I (CCTCCCACATCTGCAATGTGT) after nucleotide position 4141 and a double AT ∆ (position 4254–4255) were identified (Figure 3). This deletion has never been reported in other strains deposited in GenBank. Finally, in MCPyV 17 U only the T to A transversion in 4085 nucleotide position was found (Figure 2), whereas in plasma sample (17 P) the I (ATATTGCCTCCCACATCTGCAATGTGTCACAGGTAATATCCT), previously described for 11 P between nucleotides 4127 and 4128, (Figure 3) and three double ∆: a TG∆ (4153–4154), a CA∆ (4179–4180) and a GG∆ (4246–4247) were revealed (Figure 3).

### 2.3. Amino-Acid and Structural Analysis of MCPyV VP1

**Figure 4:**
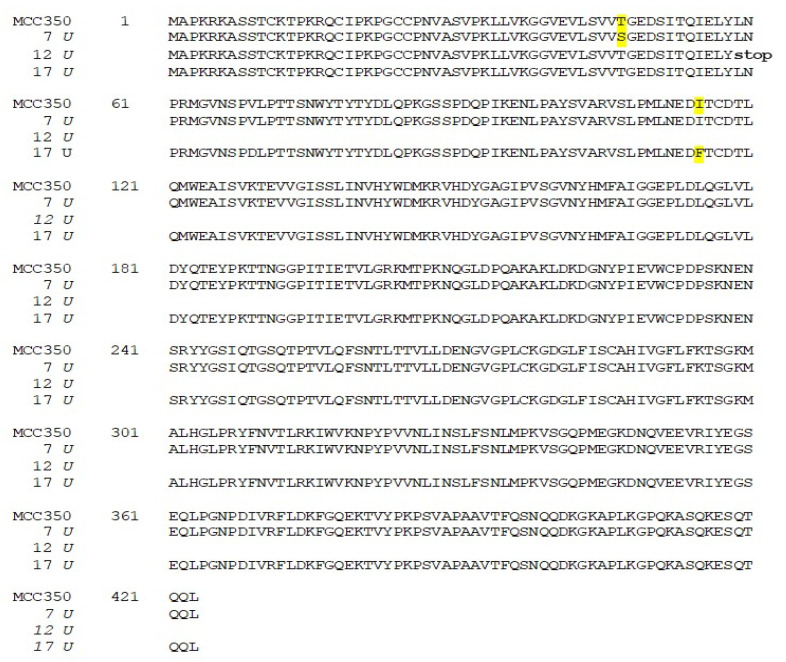
Amino-acid analysis of MCPyV VP1 in urine samples. The amino-acid translation of MCPyV VP1 nucleotide sequences revealed a VP1 characterized by Ther47Ser substitution in 7 U and by the presence of Ile115Phe substitution in 17 U (highlighted in yellow). A single nucleotide deletion in the VP1 sequence of 12 U transformed an out-of-frame stop codon into an in-frame stop codon, resulting in translation of a truncated VP1 of 58 residues.

**Figure 5:**
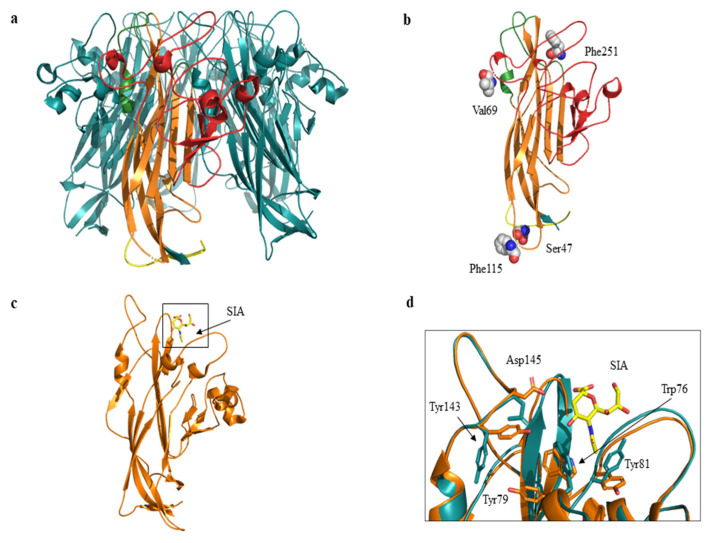
Cartoon and three-dimensional model of MCPyV VP1 pentamer and monomer. (**a**) VP1 pentamer. The monomer is highlighted in yellow (N-term region), in orange (β sandwich core), in green (apical or membrane binding loops) and in red (side or neutralizing epitope loops). The C-term region is not shown. (**b**) Three-dimensional model of VP1 monomer. Residues corresponding to the mutated sites are displayed as spheres and labelled. 7 P revealed Val69 and Phe251 substitutions, 7 U and 17 U revealed Ser47 and Phe115 substitutions respectively. (**c**) VP1 monomer shown as cartoon representation. The sialic acid (SIA) binding site is highlighted in the black box. SIA is displayed in yellow sticks. (**d**) Superposition between wild-type (orange) and ModRefiner monomer (cyan) models. Residues located in proximity of sialic acid (SIA) are displayed as sticks and labelled. Tyr79 corresponds to the mutated site.

**Figure 6:**
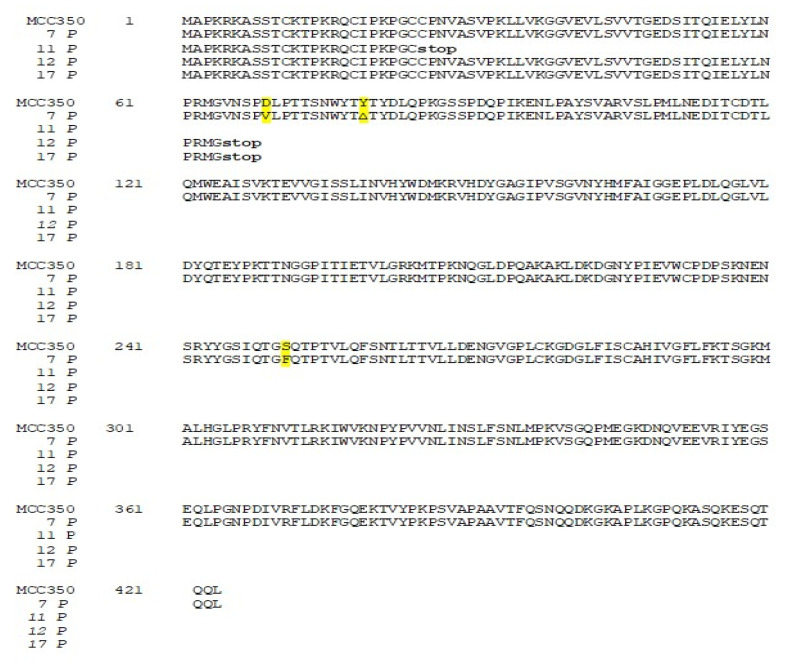
Amino-acid analysis of MCPyV VP1 in plasma samples. The amino-acid translation of MCPyV VP1 nucleotide sequences revealed a VP1 characterized by the Asp69Val, the Tyr79∆ substitutions and the Ser251Phe change in 7 P (highlighted in yellow). A stop codon did not allow the complete VP1 translation in 11 P (24 aa), 12 P (64 aa), and 17 P (64 aa).

**Figure 7:**
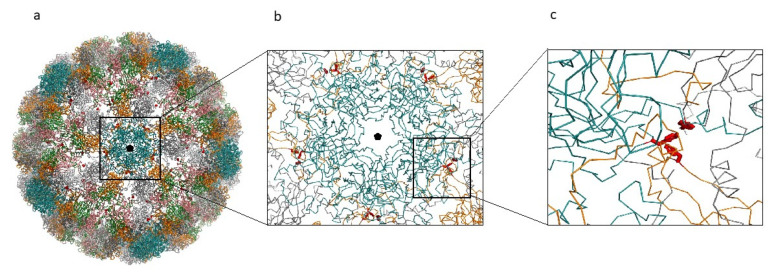
MCPyV capsid reconstruction. (**a**) MCPyV capsid reconstructed from the 6ZLZ coordinate set. The capsid is oriented with the fivefold axis of symmetry (black pentagon) perpendicular to the view. VP1 proteins of the capsid are represented as α-carbon trace and colored according to the different chains. Blue capsomers are the α-pentamers located at the vertices of the capsid icosahedron. Side-chains of the mutant Phe residues are displayed as red sticks. (**b**) Detail of the interactions between the α-pentamer and the neighboring capsomers. (**c**) Detailed view of one of the Phe clusters created by the mutation Ile115Phe.

The related amino-acid translation of MCPyV VP1 nucleotide sequences from 17 HIV-1 individuals simultaneously positive to viral DNA in plasma (MCPyV 1P-17P) and in urine (MCPyV 1 U–17 U), was also performed. As expected the analysis of 13 plasma and 14 urine (MCPyV 1 P–6 P, 8 P–10 P, 13 P–16 P and MCPyV 1 U–6 U, 8 U–11 U, 13 U–16 U) displayed a VP1 sequence structure identical to the prototype strain MCC350 (data not shown). Seven out of 34 samples (7 P, 11 P, 12 P and 17 P; 7 U, 12 U and 17 U) revealed, in two urine (7 U and 17 U), a VP1 characterized by Thr47Ser and Ile115Phe substitutions, sited in the VP1 N-terminus and in the β-sandwich core, respectively (Figure 4 and Figure 5a,b). Frame shift mutations resulted in a truncated VP1 of 58 residues in urine (12 U) and 64 residues in plasma (12 P and 17 P), (Figure 4 and Figure 6). Regarding the amino-acid analysis of the remaining samples (7 P and 11 P), 7 P revealed, within the first neutralizing epitope loop, the substitution Asp69Val, the Tyr79∆ and, located in the apical loop, the Ser251Phe change (Figure 5a,b and Figure 6). For 11 P, a nonsense mutation did not allow the complete VP1 translation and generated a truncated VP1 protein of only 24 amino acids (Figure 6). All the point mutations were exposed to the surface of the pentameric capsomer except for Tyr79∆, Thr47Ser, and Ile115Phe. Tyr79 was buried in proximity of the sialic acid binding site, situated on the surface of the pentameric capsomer, as shown in Figure 5c. Thr47Ser and Ile115Phe mutations were in a loop exposed to the internal virus capsid surface and at the interface between capsomers, respectively. Interestingly, the mutation Ile115Phe created an aromatic cluster of three Phe residues between the contact interface formed by the VP1 α-pentamer and the α’, α’’ monomers of the surrounding five capsomers [29] (Figure 7). No significant association was found between the presence of mutations versus age, gender, and HIV patient’s status (naïve/experienced). Finally, detection of mutations did not show a correlation with HIV-1 load nor with CD4+ cell counts. 

In order to predict the effect of single point mutations on VP1 structure stability, PROVEAN and DynaMut servers were employed. PROVEAN server predicted that the substitutions Asp69Val, Ser251Phe and Ile115Phe caused a neutral effect, whereas the mutations Thr47Ser and Tyr79∆ produced a deleterious effect (Table 1). The analysis conducted by DynaMut server predicted that the mutations Asp69Val, Ser251Phe and Ile115Phe stabilized the structure of VP1, whereas the mutation Thr47Ser reduced the VP1 stability (Table 1).

DynaMut was not able to predict the effect of the deletions and the consequence of Tyr79∆ was not analyzed by this method. Since Tyr79 is one of the VP1 amino acid residues situated in proximity of the sialic acid binding site, a docking method was applied to test whether the Tyr79∆ could indirectly influence MCPyV-receptor interactions. The results suggest the absence of significant variations in the ligand-protein complex. 

To gain a more accurate model refinement of the Tyr79∆ mutant, ModRefiner server was used. The superimposition of wild-type (Protein Data Bank, PDB code: 4FMG) and mutant Tyr79∆ VP1 suggests the presence of variations in the local main chain conformation and in the side chains of the residues Trp76, Tyr81, Tyr143, and Asp145 located in proximity of sialic acid (Figure 5d).

### 2.4. In Vitro Results: Replication Efficiency

**Figure 8:**
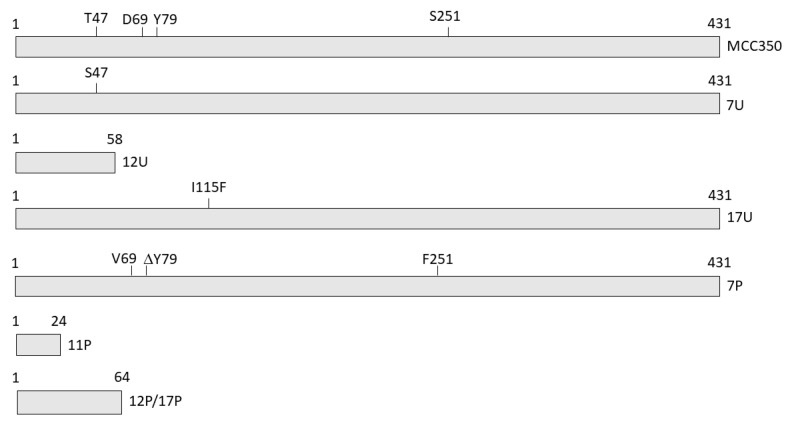
Schematic representation of the VP1 mutants detected in urine and serum from HIV1+ patients. VP1 of the prototype MCC350 is depicted on the top. Amino acid substitutions, deletion and truncated VP1 are shown.

A549 cells were used to investigate the effect of VP1 amino acid substitutions on MCPyV replication. These cells have been used earlier to study the infectious process sustained by MCPyV [26,30]. A schematic presentation of the different VP1 variants is depicted in Figure 8. 

A549 cells were infected both with supernatants (SPNTs) recovered from previous transfection experiment with MCPyV prototype DNA (MCC350 strain) [20] containing virions corresponding to 10^4^ copies/mL (considered as a viral replication control) and with urine and plasma ex vivo samples (7 U, 12 U and 17 U; 7 P, 11 P, 12 P, and 17 P) (10^4^ copies/mL). Intracellular viral DNA was extracted from 1 × 10^6^ A549 cells at different cell sampling times (3 days post infection (d.p.i.) and 7 d.p.i.) and quantified by qPCR. Results obtained from three independent experiments revealed that MCPyV DNA efficiently replicated, showing a progressively increment of viral DNA amount from 1.5 × 10^3^ copies/cell (3 d.p.i.) to 1.75 × 10^3^ copies/cell (7 d.p.i.) (Table 2). In parallel, MCPyV replication, evaluated by measuring viral DNA in the SPNTs harvested at the same cell sampling times (3 d.p.i. and 7 d.p.i.), displayed that the trend of the MCPyV load was essentially the same as that observed in the cells (data not shown). When the replication rate was evaluated in A549 cells infected with ex vivo urine (7 U, 12 U, and 17 U) and plasma samples (7 P, 11 P, 12 P, and 17 P) containing virions with mutated VP1 DNA, results showed a different efficiency of replication (Table 2). Specifically, A549 cells infected with 7 U sample carrying MCPyV VP1 characterized by Thr47Ser mutation, showed, at 3 d.p.i., a MCPyV viral load of 5 × 10^2^ copies/cell and, at 7 d.p.i., a MCPyV viral load of 9.2 × 10^2^ copies/cell (Table 2). A549 cells infected with samples 12 U and 12 P, characterized by virus genomes encoding truncated VP1, did not produce a detectable viral replication (Table 2). Finally, A549 cells infected with 17 U sample, carrying Ile115Phe substitution, located in the VP1 β-sandwich core, showed, at 3 d.p.i., a MCPyV viral load of 1 × 10^3^ copies/cell and, at 7 d.p.i., of 1.2 × 10^3^ copies/cell (Table 2). The MCPyV replication in A549 cells infected with plasma samples was observed only in cells infected with 7 P, despite the presence of Asp69Val, Tyr79∆ and Ser251Phe. In detail, at 3 d.p.i., the MCPyV viral load was of 1 × 10^2^ copies/cell and, at 7 d.p.i., of 1.2 × 10^2^ copies/cell (Table 2). The remaining plasma samples (11 P and 17 P) were unable to infect A549 cells (Table 2). The trend of the MCPyV load in the SPNTs, was essentially the same observed in cells infected with 7 U and 17 U and with 7 P (data not shown). A549 cells exposed to 12 U, 12 P, 11 P, and 17 P samples showed a low amount (< 100 copies/mL) of viral DNA (data not shown), which possibly represented the DNA present in the urine and plasma specimens incapable to produce a productive infection. In order to confirm and support the findings that viral particles were generated during the first infection, SPNTs, collected during in vitro experiments, were used to infect freshly seeded A549 cells. MCPyV replication was evaluated in A549 cells used as a control in which the viral amount of DNA was at 3 d.p.i. of 2 × 10^3^ copies/cell and, at 7 d.p.i., a MCPyV load of 3.5 × 10^3^ copies/cell (Table 2) and in cells infected with SPNT 7 U, carrying a VP1 characterized by Thr47Ser. In this sample, at 3 d.p.i. and at 7 d.p.i., a viral load of 8.5 × 10^2^ copies/cell and of 1.5 × 10^3^ copies/cell was respectively detected (Table 2). Infection using SPNT 17 U with Ile115Phe substitution, displayed a MCPyV load of 5.8×10^3^ copies/cell at 3 d.p.i. and of 9 × 10^3^ copies/cell at 7 d.p.i. (Table 2). Finally, A549 cells infected with SPNT 7 P in which Asp69Val, Tyr79∆, and Ser251Phe were detected, evidenced at 3 d.p.i., a viral load of 6.2 × 10^2^ copies/cell and, at 7 d.p.i., of 1 × 10^3^ copies/cell (Table 2). The remaining SPNTs (SPNT 12 U, SPNT 11 P, SPNT 12 P, and SPNT 17 P) carrying truncated VP1 were unable to infect A549 cells de novo (Table 2). The levels of viral DNA increase from 3 d.p.i. to 7 d.p.i. during both the first and second cycle of infection. 

MCPyV replication was also evaluated by measuring viral DNA in the SPNTs obtained during the second round of infection. Results confirmed the same MCPyV replication trend observed in the first cycle of infection and the lack of viral replication in SPNTs obtained from cells infected with SPNT 12 U, SPNT 11 P, SPNT 12 P, and SPNT 17 P (data not shown).

### 2.5. In Vitro Results: Immunofluorescence Staining

**Figure 9:**
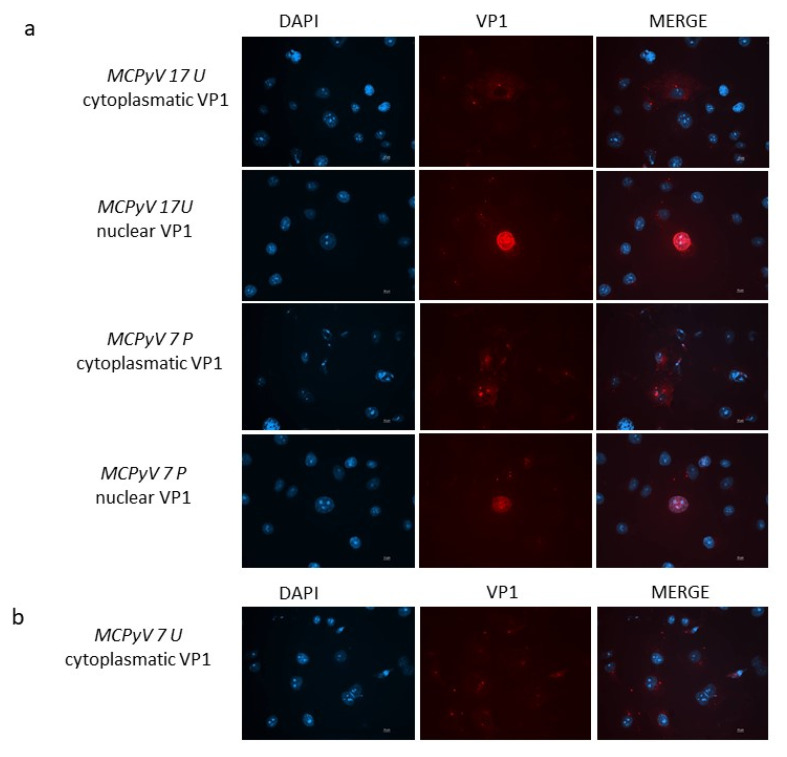
(**a**) VP1 expression in A549 cells. Immunofluorescence staining with VP1 antibodies was conducted at 7 d.p.i. in cells infected with MCPyV prototype MCC350 strain, 17 U or 7 P samples. (**b**) Immunofluorescence (IF) conducted at 7 d.p.i. in cells infected with 7U. DAPI staining (blue color) was performed to show the nucleus. Scale bar, 10 μm.

In order to attest whether viral progeny was produced during infection experiments, the intracellular localization of MCPyV VP1 was monitored by immunofluorescence (IF) experiments. At 3 d.p.i. MCPyV VP1 was not detected neither in cells infected with the prototype DNA nor cells infected with ex vivo samples. When IF was conducted at 7 d.p.i., cells infected with either the MCC350 strain, 7 U, 17 U, or 7 P samples, all revealed MCPyV VP1 expression. In detail, A549 infected with 17 U and 7 P showed both cytoplasmic and nuclear localization of MCPyV VP1 (Figure 9a). A459 cells infected with 7U displayed only cytoplasmic localization of the protein (Figure 9b). MCC350 expressing VP1, can be considered the positive control of the experiments whereas IF, performed on all mutants unable to replicate, did not reveal a VP1 production, representing the negative control. IF staining on A549 cells that were not challenged with MCC350 or urine or plasma samples did not result in any staining (results not shown).

### 2.6. In Vitro Results: VP1 Amplification, Analysis and Sequencing

The nucleotide and the corresponding amino-acid analysis of samples recovered from A549 cells confirmed the presence of a VP1 structure characterized by the same mutations observed in ex vivo samples.

## 3. Discussion

MCPyV is the first HPyV directly implicated in the development of a specific cancer, namely MCC. Although MCPyV is constantly shed from healthy skin and up to 60–80% of healthy people have antibodies against this virus, MCC is rare with an incidence of 0.2–0.45 cases/100,000 individuals per year in most countries [13]. The MCC incidence increases with aging [31,32,33,34,35] and among immunocompromised individuals such as HIV-1/AIDS patients [15,35,36,37,38]. To date, the events connecting initial MCPyV infection and subsequent cell transformation remain elusive, although it has been established that viral DNA integration into the host genome and continued expression of the C-terminal truncated LT are required for MCC development [20]. The C-terminus of LT contains anti-tumorigenic properties and explains why this region is deleted in MCC [12]. In addition to LT truncation, also mutations in VP1 have been proposed to be indirectly implicated in viral DNA integration and tumorigenesis [21,22]. As known, VP1 is the major capsid protein involved in the mechanisms underlying protein self-assembly, attachment of HPyVs to host cell receptors, virus entry, immune response and intracellular trafficking [23,24,27]. Moreover, VP1 plays an important role in serological diagnosis of HPyVs infection and in epidemiologic studies [39]. The three-dimensional structure of MCPyV VP1 has shown that it is a symmetric ring-shaped homopentamer with the five monomers arranged around a central five-fold axis. Each VP1 monomer is composed of two antiparallel β-sheets, which together form a β-sandwich with jelly-roll topology. The β-strands are connected by loops on the top and the sites of the pentamer. These loops are the most variable parts of VP1 sequences [40]. Mutations within the VP1 could be considered as key events in modifying tissue tropism, virulence, and subsequent infections [23,24,25,26,27]. Given the limited data on the genetic variability of MCPyV VP1, in this study, the properties of this protein, circulating among a HIV+ population, and its potential effects on protein structural organization, were investigated. The overall analysis of VP1 sequences recovered in this study, showed a high degree of identity respect to the prototype strain MCC350. None of the nucleotide mutations detected in our MCPyV VP1 DNA sequences have been reported before and, consequently, also the amino-acid mutations had never been reported previously, except for the Ser251Phe change, described by Baez and colleagues [21]. The substitution Ser251Phe occurred in the apical or membrane-binding loop, considered responsible for virus-cell interaction [41]. 

Although no significant association was found between MCPyV viral load and the onset of mutations versus age, gender, HIV patient’s status (naïve/experienced), HIV-1 load and CD4+ cell counts, the higher number of substitutions and ∆ occurred in 7 U and in 7 P. Interestingly, this urine and plasma sample belonged to a naïve individual. The weakened immune system with low CD4+ counts, representative of naïve status, might favor MCPyV replication resulting in VP1 variability. This observation needs to be further explored.

MCPyV, in contrast with other PyVs such as SV40, BKPyV, and JCPyV, which use carbohydrates containing sialic acid for cell attachment and internalization [42,43,44], utilizes sulfated carbohydrates, termed glycosaminoglycans (GAGs), as attachment receptors and sialic acids in the post-attachment process [25,30]. Specifically, MCPyV for cell attachment and internalization recognizes the N-acetyl neuraminic acid (Neu5Ac) binding site motif, situated in the apical loop [41]. It has been previously demonstrated that mutations in or near the Neu5Ac binding site limit or abolish the MCPyV ability to infect A549 cells [41]. In addition to the Ser251Phe change within the first neutralizing epitope loop in the MCPyV 7 P sample, the substitutions Asp69Val and the Tyr79∆ were observed [40]. Since no analysis to investigate MCPyV immune escape was performed in this study, it is exclusively possible to speculate that these two mutations could concur to limit the antibody response against MCPyV that is typically generated versus epitopes exposed at the surface of the VP1. While SV40, BKPyV and JCPyV have the consensus sequence GXPD (with D corresponding to Asp69 in MCPyV), MCPyV VP1 has GVNSPD and HPyV10 and STLPyV have GPD. The role of Asp69 is not known, but it lies adjacent Neu5Ac binding site, that, as described above, represents a major cell surface interaction compound for MCPyV VP1 [41]. Mutation of D60N (that corresponds with D69 in MCPyV VP1) in combination with K69N, A72V and E82Q in BKPyV VP1 reduced BKPyV infectivity in HEK293TT cells 5-fold compared to BKPyV with wild-type VP1 [45]. The D60N mutation in BKPyV VP1 disrupts the epitope bound by the neutralizing monoclonal 41F17, but the effect of the single D60N mutation on BKPyV infectivity was not tested [46]. Whether D69 in MCPyV VP1 of virus particles is part of an epitope remains elusive. The sequence encompassing Tyr79 does not show similarity with VP1 of other HPyVs except that Tyr is followed by Ser or Thr in VP1 of MCPyV, TSPyV, HPyV9, HPyV10, STLPyV, HPyV12, NJPyV, and LiPyV. The functional importance of Tyr79 and the adjacent S/T is not known. On the contrary, amino-acids ∆ in structural protein of HPyVs and also other human DNA viruses like human papillomavirus are well known and mutations in their capsid proteins have been proposed to be involved in the process of viral DNA integration or as a consequence of this process [21,22,47,48,49]. In this framework, to address the consequences of the VP1 mutations, in vitro experiments were performed in order to understand whether the amino-acids changes observed in this study, could have any implications on MCPyV biology. MCPyV replication was assessed in A549 cells. These cells have been used earlier to study infectious process sustained by MCPyV [26,30]. A549 cells were infected with MCPyV 7 P sample, in which Ser251Phe was detected in concomitant with the substitution Asp69Val and Tyr79∆. Results obtained by qPCR assay, showed that viral replication is less than 1-log for MCPyV 7 P sample compared to samples infected with MCPyV prototype DNA (positive control). These data allow supposing that different amounts of viral DNA entered the cell, highlighting the relevance of site near to Neu5Ac for MCPyV entry into the host cell. Further study will be needed to determine the state of the viral genome (episomal or integrated) carrying amino acids ∆ in VP1. Only one mutation, the Thr47Ser, was found sited in the VP1 N-terminus region. This change in the VP1 of urine specimen 7U is located in a region that corresponds with the AB loop of SV40 VP1 [50]. The sequence of this loop is VKTGVD and, a similar motif with consensus VX_0–3_(T)(S)X_1–2_(D)(E), is found in BKPyV, JCPyV, KIPyV, WUPyV, MCPyV, HPyV6, HPyV7, TSPyV, HPyV9, NJPyV, and LiPyV. MCPyV has the sequence VVTGED (Thr47 is underlined). In vitro replication experiments revealed that A549 cells infected with 7 U sample, carrying MCPyV VP1 characterized by Thr47Ser mutation, supported MCPyV replication less well than A549 cells infected with MCPyV prototype DNA (MCC350 strain) and containing virions. This trend occurred in both the first and second round of infection experiments. The Ile115Phe substitution in MCPyV VP1 of sample 17U, sited in the VP1 β-sandwich core, is located in the corresponding CD-loop of SV40 VP1 and is part of the consensus sequence ED(L)(I)(M)T found in VP1 of SV40, BKPyV, JCPyV, TSPyV, HPyV10, HPyV12, and LiPyV. Ile115Phe, found into the β-sandwich region, near to the two β-sheets, could prejudice the stability of the β-sandwich core and subsequently the whole VP1 protein. The trend of the MCPyV load in A549 cells infected with virions characterized by Ile115Phe was essentially the same observed in cells infected with MCPyV prototype DNA (control), confirming that this mutation is not involved in attachment of the virus to host cell receptors, virus entry or in its replicative capabilities. Except for the Ile115Phe substitution, all the variations detected in this study are involved in epitopes recognized by B cells. None of these mutations are involved in epitopes recognized by T cells [51]. Regarding the protein stability, in order to predict the effect of single point mutations on VP1, structure-based analysis was performed by PROVEAN and DynaMut servers. Both servers found that the variant Ile115Phe, sited near to the two β-sheets, into the β-sandwich region, did not affect the conformation, but stabilizes the protein structure. The mutation creates an aromatic cluster of three Phe at each vertex of the apical pentameric capsomer. It may be speculated that the combination of the effects of the single clusters can produce a significant stabilization of the entire capsid. The DynaMut server analysis suggests that the mutation Thr47Ser reduces protein stability. This result was further validated by the PROVEAN server analysis, which also envisages a destabilizing effect, although none of the two methods predict a highly deleterious effect on the structural stability of VP1 protein. By DynaMut, it was not possible to predict the effect of the Tyr79∆ on the protein stability. However, the deletion removes a bulky residue that resides in the proximity of the sialic acid binding site. The superimposition of the wild-type and the model of the mutant Tyr79∆ suggests the presence of a local change of the main chain conformation. Although docking experiments have not displayed any significant alteration of the binding, this aspect may be worthy of further investigations. 

Four samples, 11P, 12P, 17P, and 12U, contained VP1 sequences encoding C-terminally truncated VP1 of 24, 124 (VP1 sequences of 12P and 17P were identical) and 58 amino acids, respectively. The truncated proteins are unable to form infectious virions and probably represent defectively replicated genomes released from infected cells. In order to attest whether viral progeny was produced during infection experiments, the intracellular localization of MCPyV VP1 was monitored by IF experiments. At 3 d.p.i. MCPyV VP1 was not detected neither in cells infected with the prototype MCC350 strain (positive control) nor cells infected with ex vivo samples. This can be explained by assuming that, as demonstrated in previously studies with MCPyV pseudo viruses, A549 cells are highly susceptible for MCPyV attachment although viral entry is slow and plateaus after 72 h [25,30]. When IF was conducted at 7 d.p.i., cells infected with prototype MCC350 (positive control) and with 7 U, 17 U, and 7 P samples revealed MCPyV VP1 expression. In detail, A549 cells infected with 17 U and 7 P showed an intracellular localization of MCPyV VP1, both in the cytoplasm and in the nucleus. VP1 was efficiently transported into the nucleus where viral assembly normally occurs. On the contrary, A459 cells infected with 7U displayed only cytoplasmic localization of the protein. The mechanism of VP1 transport through the cytoplasm towards the nucleus is due to the presence of the nuclear localization signal (NLS) of VP1 in its N-terminus sequence. Although the Thr47Ser substitution is sited in the VP1 N-terminus region, this mutation did not compromise the NLS sequence and did not prevent the transfer of VP1 from the cytoplasm to the nucleus. Nuclear import of VP1 is needed for the correct replication of the virus. When the replication rate was evaluated in A549 cells infected with ex vivo urine 7, replication efficiency was demonstrated. Consequently, the lack of nuclear visualization did not depend on the lack of transport to the nucleus, but it is possible that the whole virus assembly process is delayed in 7U infected cells and/or that little VP1 is in the nucleus and, therefore, not detected. This is the first study in which it has been demonstrated that A549 cells can support MCPyV infection, as proven by the increase in viral genome copies from 3 d.p.i. to 7 d.p.i., a full MCPyV replication cycle occurred, as showed by the expression of VP1, and supernatants from primary infected cells were able to re-infect freshly A549 cells. In conclusion, in this study we observed mutations that are unique, indicating the variability in VP1 of HIV-1 + individuals and developed a (semi)-permissive A549 culture system that allows to investigate the consequences of VP1 genetic variability on MCPyV replication. 

Since previous studies showed that persistent high-level BKPyV viruria in renal transplant recipients is associated with accumulation of mutations in VP1 [45,52], longitudinal studies to investigate whether mutations accrue in MCPyV VP1 in individuals with persistent or intermittent shedding of MCPyV should be performed. Moreover, in this study, PCR was used to amplify MCPyV VP1 sequences in the biological samples. This method will amplify the most prominent variant present in the specimen, whereas deep sequencing allows identification of less represented sequences. For example, next generation sequencing was successfully applied to identify several JCPyV strains and mutations in the VP1 gene in urine from a kidney transplant patient [53] and in cerebrospinal fluid from Progressive Multifocal Leukoencephalopathy patients [54]. A similar approach could be used to detect other variants in the same samples from HIV-1 positive individuals.

## 4. Materials and Methods

### 4.1. Study Population

A cross-sectional study including a cohort of 100 HIV-1-positive individuals, admitted to the Infectious Diseases Clinic of the Polyclinic Tor Vergata Foundation from January 2019 to February 2020, was performed. Among the enrolled patients, 40 were new diagnoses naive to treatment and 60 were experienced patients on treatment with a triple-based antiretroviral regimen including protease/reverse transcriptase/integrase inhibitors. From this cohort (76 males/24 females, age ranged from 21 to 76 years old: mean age ± standard deviation: 40.5 years old; median: 39.9 years old), a urine and plasma sample from each patient were collected. In detail, 100 plasma and 100 urine were obtained for a total of 200 specimens. Demographic and clinical characteristics are presented in Table 3. The study was approved by the local Ethic Committee of the University Hospital Tor Vergata (Rome, Italy) (protocol number 0027234/2018, 19 December 2018), and informed consent was obtained from patients.

### 4.2. MCPyV DNA Extraction and Quantification by qPCR 

Total DNA was extracted from urine and plasma using the DNeasy^®^ Blood & Tissue Kit (QIAGEN, Milan, Italy), according to the manufacturer’s instructions. Specific qPCR assays were performed using TaqMan-based qPCR, employing primers and probes for MCPyV sT, as previously described [35]. All samples were tested in triplicate, and the number of viral copies was calculated from standard curves constructed using a ten-fold dilution series of plasmid pMCV-R17a containing the entire genome of MCPyV (Addgene, #24729) (dilution range: 10^8^–10 copies/mL). The lower detection limit of the assay was 10 DNA copies of the target gene per amplification reaction, corresponding to 10 copies per reaction (10 copies/reaction). The amount of cellular DNA was quantified simultaneously using a SYBR GREEN PCR for the housekeeping β-globin gene and used to normalize the MCPyV DNA. The processing of all samples was accomplished following the technical guidelines on laboratory biosafety.

### 4.3. Sample Selection and VP1 Amplification, Analysis, and Sequencing

Samples simultaneously positive to MCPyV DNA detection in urine and plasma were arbitrarily selected and used for MCPyV VP1 amplification and sequencing. The MCPyV *VP1* gene was amplified through a nested PCR using two different primer sets generating two fragments of 494 bp and 307 bp, respectively [55]. The 307 bp resulting fragments were analyzed on 2% agarose gels by ethidium bromide staining, purified using the MinElute PCR Purification Kit (QIAGEN, Milan, Italy) and confirmed by sequencing, using sense and antisense primers, by a dedicated facility (Bio-Fab Research, Roma, Italy). Sequences were generated using the Big Dye Terminator Sequencing method (Life Technologies, Carlstad, CA, USA) on the ABI 3730 sequencer (Life Technologies), and analyzed with the Sequencing Analysis 5.2 software (Life Technologies). Sequence alignments were conducted with ClustalW2 at the EMBL-EBI website using default parameters [56]. 

### 4.4. VP1 Amino-Acid Translation and Analysis

The MCPyV VP1 nucleotide sequences obtained from plasma and urine samples were translated into amino-acid sequences using the Blastx [57] and EMBOSS Transeq [58] programs and were aligned using the Jalview software [59]. Homology modelling relied on Modeller [60] and structure display and analysis on PyMOL [61]. The structure of the variant forms of VP1 were obtained by homology modelling using as a template the structure of the wild-type protein deposited in PDB as Merkel cell polyomavirus VP1 (PDB code: 4FMG and 6ZLZ). The VP1 structure can be divided into six functional regions: N-terminus (residues 41–51), β-sandwich core with jelly-roll topology (residues 52–59, 102–132, 161–169, 241–248, 254–295, 298–316), apical or membrane binding loops (residues 133–160, 249–253, 296–297), side or neutralizing epitope loops (residues 60–101, 170–240) and conserved region of the C-terminus (residues 316–320). The models were constructed without 1–40 residues of N-term region and without C-term domain (321–423 residues). The prediction of the impact of single point mutation on the stability of the VP1 protein was performed using in silico methods such as PROVEAN and DynaMut servers [62,63]. Docking method implemented in AutoDock Vina was applied to evaluate whether the Tyr79∆ mutation may influence the interaction between VP1 and sialic acid [64]. Interactions between monomers belonging to different pentameric capsomers were studied using the reconstructed virus capsid available in the Protein Data Bank (PDB) as the biological unit of 6ZLZ structure. The capsid has been reconstructed starting from the coordinate set 6ZLZ with application of icosahedral symmetry operations. Model refinement was applied using ModRefiner server [65].

### 4.5. In Vitro Cells Cultures: Transfection, Infection, and Evaluation of the Replication Efficiency

The non-small-cell lung cancer cell line A549 [66] was used to perform the in vitro experiment. MCPyV DNA was recovered using *BamH*I-digested pMCV-R17, a plasmid containing the entire genome of MCPyV prototype strain MCC350 (Addgene, #24729). The obtained viral linear DNA was gel-extracted, purified using a GenepHlow TM DNA Cleanup Maxi Kit (Geneaid Biotech Ltd., New Taipei City, Taiwan) and then quantified. One μg of MCC350 strain DNA was used to transfect A549 cells (10^4^), following the specifications of the Xfect TM Transfection Reagent kit (Clontech Laboratories, Inc., Mountain View, CA, USA). Cells were incubated at 37 °C with the transfection mixture and after 4 h washed with phosphate-buffered saline (PBS). Subsequently, cells were incubated with complete culture medium until 15 days post-transfection (d.p.t.). SPNTs collected two times a week during transfection experiments, were subjected to 6 cycles of freezing and thawing, followed by centrifugation at 2000 rpm for 5 min [67]. The resulting clarified SPNTs were quantified using TaqMan-based qPCR, employing primers and probes for MCPyV sT, as previously described [35] and virions corresponding to 10^4^ copies/mL were used to infect freshly seeded A549 cells. Cells and SPNTs infected with MCPyV prototype DNA were used as controls during the experiments with ex vivo plasma and urine samples. In parallel, also MCPyV plasma and urine positive samples, recovered from HIV+ individuals (MCPyV 7U, 11U, 12U, 17U, and MCPyV 7P, 11P, 12P, 17P) and displaying a VP1 structure characterized by the presence of several amino-acids mutations, were subjected to MCPyV DNA quantification by qPCR and 10^4^ copies/mL used to infect A549 cells. After 2 h of adsorption, cells were washed 3 times with PBS and incubated with fresh medium. At established time points (3 d.p.i. and 7 d.p.i.), cells and SPNTs were collected and viral DNA was extracted from 1 × 10^6^ A549 cells using a QIAmp^®^ DNA Mini Kit (QIAGEN S.p.A., Milan, Italy), following the instructions provided by the manufacturer. SPNTs from A549 cells were used directly in molecular biology assays. Extracted DNA and SPNTs were quantified using qPCR for MCPyV sT, [35]. The amount of cellular DNA was quantified simultaneously using a SYBR GREEN PCR for the house-keeping β-globin gene [68] and used to normalize the MCPyV DNA. The data were expressed as copies of viral DNA per cell based on DNA content (copies/cell) for the A549 cells and as copies of viral DNA per milliliter (copies/mL) for the supernatants. All of the in vitro cells experiments were accomplished following the technical guidelines on laboratory biosafety.

### 4.6. In Vitro Cells Cultures: Immunofluorescence Staining

IF was carried out for VP1 subcellular localization. Infected and uninfected A549 cell monolayers were fixed with 4% paraformaldehyde and permeabilized for 20 min with a 0.5% solution of Triton X-100 in PBS at RT for 1 h. Cell monolayers were first incubated with a rabbit polyclonal anti-MCPyV VP1 antibody (1:2000 dilution; Alexa Fluor), and afterwards with an anti-rabbit IgG secondary antibody (1:500 dilutions; Alexa Fluor) [69]. Cells were washed with PBS and cellular DNAs were labelled with 4′, 6′-diamidino-2-phenylindole (DAPI, Molecular Probes). Single images were acquired with a Leica DM5000B microscope equipped with the Digital FireWire Color and Black and White Camera systems LeicaDFX350 and DFX300, respectively, and processed using the Leica Application Suite 2.7.0.R1 software (Leica).

### 4.7. In Vitro Cells Cultures: Analysis and Sequencing of MCPyV VP1

VP1 amplification, analysis and sequencing of samples recovered from cells were performed as described above.

### 4.8. Statistical Analysis

MCPyV detection was summarized by counts and proportions. If continuous variables were normally distributed, they were expressed as mean ± SD; if not, they were expressed by median and range. The χ2 test was performed to evaluate differences for categorical variables. Group differences for continuous variables were tested using Student’s t-test or Mann–Whitney U-test, for normally and non-normally distributed variables, respectively. Associations between two continuous variables were examined by Pearson or Spearman correlation for normally or non-normally variables. Differences in viral loads, among the three anatomical sites, were analyzed using Kruskal-Wallis test. A *p*-value less than 0.05 was considered statistically significant.

## 5. Conclusions

This study shows that MCPyV variants with mutations in VP1 compared to the MCC350 prototype are circulating in plasma and urine of HIV-1-positive individuals. Protein stability prediction programs foresee that the detected amino acid substitutions D69V, I115F, and S251F stabilize the VP1 protein, while the T47S mutation is suspected to destabilize the protein. Infection studies in A549 cells suggest that the mutant strains display a replication efficiency comparable with MCC350, except for the T47S mutant. The effect of these mutations on the pathogenic properties of the virus and the immunogenicity of VP1 remains to be examined.

## Figures and Tables

**Table 1:** Comparative studies by PROVEAN and DynaMut for protein stability prediction.

Variants	PROVEAN Score; Effects	DynaMut; Effects
Asp69Val	−1.994; neutral	ΔΔG: 0.506 kcal/mol; stabilizing
Ser251Phe	−1.459; neutral	ΔΔG: 1.431 kcal/mol; stabilizing
Ile115Phe	−1.809; neutral	ΔΔG: 1.371 kcal/mol; stabilizing
Thr47Ser	−2.586; deleterious	ΔΔG: −0.442 kcal/mol; destabilizing
Tyr79∆	−4.793; deleterious	

PROVEAN score cut-off = −2.5; ∆∆G: structural stability.

**Table 2:** Replication efficiency in A549 as determined by qPCR.

**1st Round of Infection**			
**MCPyV Variant**	**3 d.p.i.**	**7 d.p.i.**	Increase (%) *
MCC350	1.5 × 10^3 #^	1.75 × 10^3^	17
7U	0.5 × 10^3^	0.92 × 10^3^	84
12U	-	-	-
12P	-	-	-
11P	-	-	-
17P	-	-	-
17U	1 × 10^3^	1.2 × 10^3^	20
7P	0.1 × 10^3^	0.12 × 10^3^	20
**2nd round of infection**			
**MCC350**	**2 × 10^3^**	**3.5 × 10^3^**	**75**
7U	0.85 × 10^3^	1.5 × 10^3^	76
12U	-	-	-
12P	-	-	-
11P	-	-	-
17P	-	-	-
17U	5.8 × 10^3^	9 × 10^3^	55
7P	0.6 × 10^3^	1 × 10^3^	67

^#^ viral genome copies/cell; * increase in viral genome copies from 3 d.p.i. to 7 d.p.i.; -no replication.

**Table 3:** Demographics and clinical features of HIV-1 positive individuals at enrolment.

Patients Enrolled	Total *n*.	Gender, *n*.	Age	HIV-1 RNA Load, Range	CD4+ Counts, Range
**Naive**	40	Male: 30; Female: 10	Range: 21–68 y.o. Mean: 39.27 y.o.	1.26 × 10^3^–10 × 10^6^ copies/ml	9–890/mm^3^
**Experienced**	60	Male: 46; Female: 14	Range: 21–76 y.o. Mean: 43.7 y.o.	TND *-27 × 10^5^ copies/ml	312–1178/mm^3^

*n*: number; y.o: years old; TND: Target not detected; * increase in viral genome copies from 3 d.p.i. to 7 d.p.i.

## Abbreviations

MCPyV	Merkel Cell Polyomavirus
T	Tumor
LT	Large T
sT	small T
ALTO	LT open reading frame
VP1	viral protein 1
VP2	viral protein 2
ATG	adenine-thymine-guanine
HPyVs	human polyomaviruses
NCCR	non-coding control region
ORI	origin of replication
NLS	nuclear localization signal
VLPs	virus-like particles
MCC	Merkel cell carcinoma
qPCR	quantitative Real-Time polymerase chain reaction
IF	immunofluorescence
SPNTs	supernatants
I	insertion
∆	deletion
d.p.t.	days post-transfection
d.p.i.	days post infection
PBS	phosphate-buffered saline
